# Primary desmoplastic small round cell tumor of the testis: A case report and review of the literature

**DOI:** 10.3892/ol.2013.1421

**Published:** 2013-06-25

**Authors:** LANG HE, SHIMIN WEN, XIN HU, CUIHUA GUO, CHENG YI

**Affiliations:** 1 Department of Abdominal Cancer, Cancer Center of West China Hospital, West China Medical School, Sichuan University, Chengdu, Sichuan 610041, P.R. China; 2 Cancer Center, the Second Clinical Medical College of North Sichuan Medical College, Nanchong Central Hospital, Nanchong, Sichuan 637000, P.R. China

**Keywords:** desmoplastic small round cell tumor, testis

## Abstract

Desmoplastic small round cell tumors (DSRCTs) are extremely rare and mainly affect adolescents and young adults. The tumors are usually involved with the abdominal area and/or the pelvic peritoneum. Only a small number of cases have been reported concerning DSRCTs of the testicular region. The present study reports a case of DSRCT of the testis with radical orchectomy and systemic chemotherapy, leaving the patient disease-free for 14 months. However, the patient died of multiple metastasis 12 months later. Furthermore there is a review of the English literature to analyze the incidence, site of origin, imaging and pathological characteristics of DSRCT.

## Introduction

A DSRCT is an aggressively malignant tumor that predominantly occurs in adolescents and young adults. The condition was first described by Gerald and Rosai in 1989 (1) and usually arises in the abdominal area and/or the pelvic peritoneum, presenting with a diffuse peritoneal extension. Extra-abdominal DSRCTs, particularly those arising in the testis are rare. To the best of our knowledge, only one study has been published with regard to a DSRCT of the paratesticular region (2). The present study describes an unusual case of DSRCT in a Chinese patient, and may be the first primary DSRCT of the testes to be reported in the English literature.

## Case report

**Figure 1.**
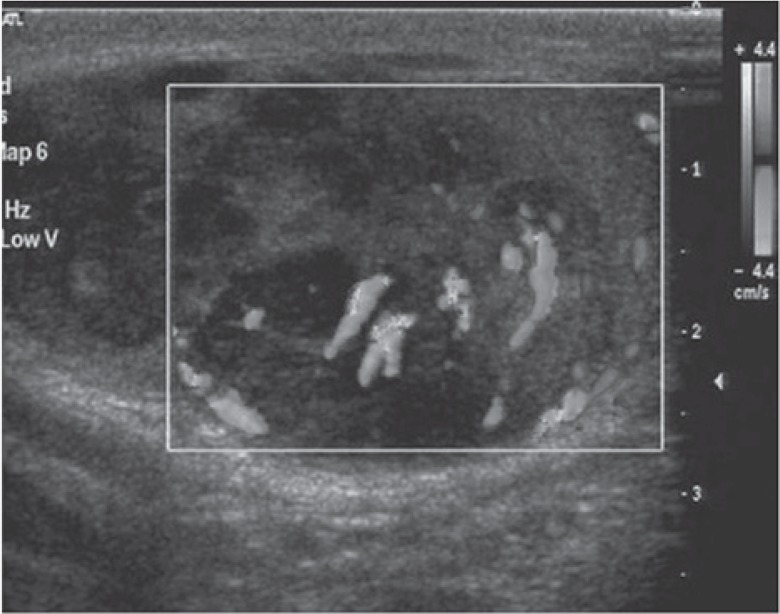
Doppler sonography image of the right testicular region demonstrating a large mass with mixed echogenicity and blood flow signal.

**Figure 2.**
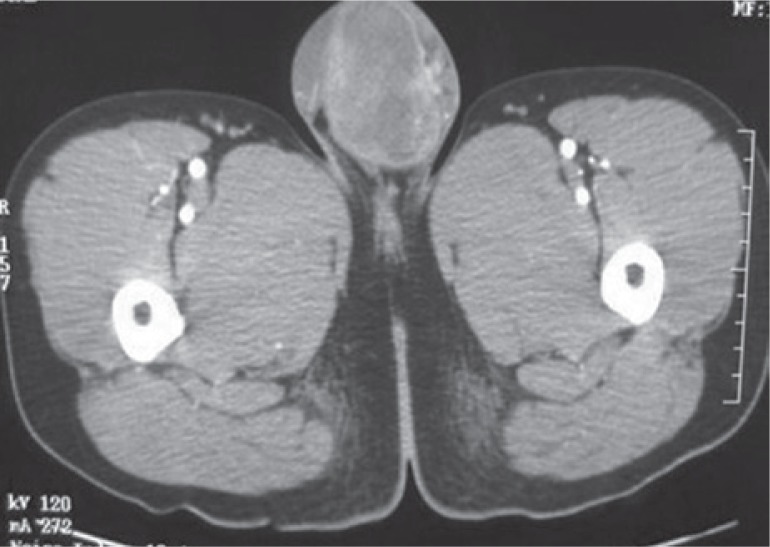
CT scan of the scrotum demonstrating a large hyperdense mass measuring ∼5.5×5 cm in the right testicular region. CT, computed tomography.

A 27-year-old male presented with gradual swelling and intermittent testicular pain that had lasted for approximately four months. There was no specific infection or a history of trauma. The patient was initially diagnosed with epididymitis in a clinic and treated with antibiotics for two weeks. However, no significant improvement in the condition was observed. A physical examination revealed a solid mass located in the right scrotum, with no tenderness. Laboratory studies did not reveal any abnormalities. A mass measuring ∼5×6 cm (Fig. 1) was identified in the right testis using ultrasound sonography. A computed tomography (CT) scan revealed a solitary mass of high intensity in the right testis, with a regional extension to the epididymis. A low-density area was identified inside the mass (Fig. 2). There was no evidence of metastasis to the local or distant organs. The patient provided written informed consent.

**Figure 3.**
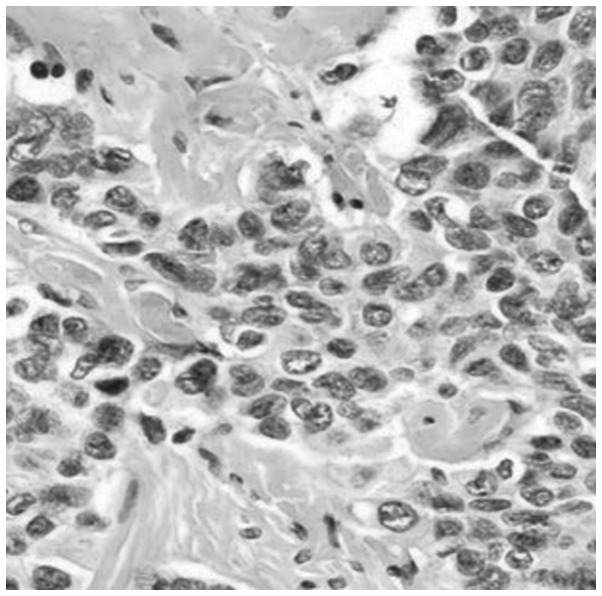
Small tumor cells with round to oval nuclei and a small amount of cytoplasm (hematoxylin and eosin staining; original magnification, ×400).

**Figure 4.**
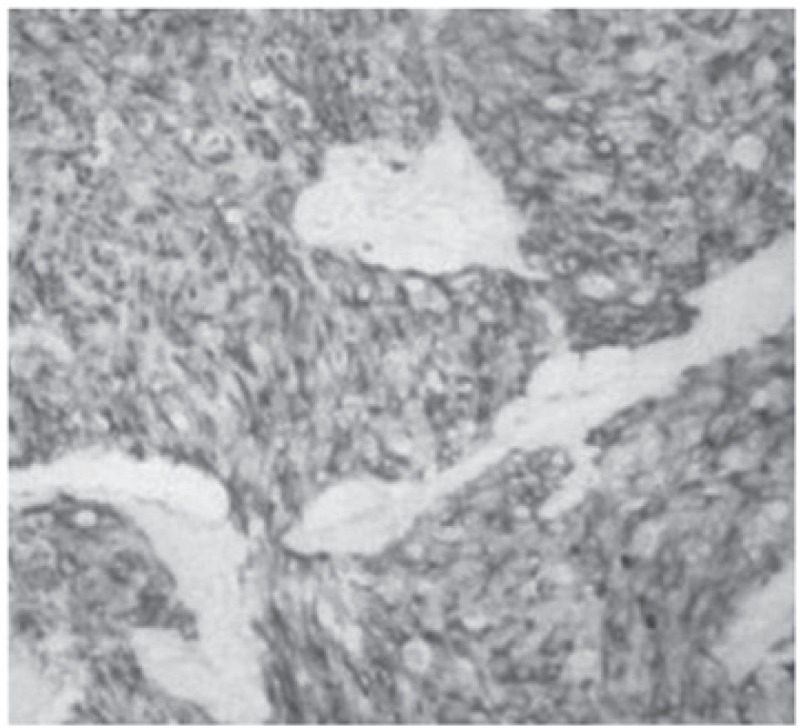
Tumor cells forming well-delineated nests that are positive for neuron-specific enolase (NSE). (Immunohistochemical analysis; original magnification, ×100).

The patient was diagnosed with a malignant tumor and a radical orchectomy was performed. The post-operative course was smooth. A formalin-fixed, paraffin-embedded tissue section was obtained for a routine microscopic examination. The specimen was stained with hematoxylin and eosin. Microscopically, the tumors consisted of nests of ‘small cells’, with scant cytoplasm embedded in a densely fibrotic stroma and focal tubule formation. Numerous mitotic figures were observed within the tissues. Certain figures were arranged in well-defined cell nests, which were delimited by a cellular desmoplastic stroma (Figs. 3 and 4). Immunohistochemical staining was performed using the streptavidin-biotin peroxidase method. Immunohistochemically, the tumor cells were positive for smooth muscle actin (SMA), vimentin, CD99 and neuron-specific enolase (NSE). However, the cells were non-reactive for Human Melanoma Black-45 (HMB45) and cytokeratin (CK). The histological and immunohistochemical findings supported the diagnosis of a desmoplastic small round cell tumor (DSRCT). The patient was administered treatment consisting of a multi-agent systemic chemotherapy regimen every three weeks in four cycles with 1.4 mg/m^2^ vincristine on the first day, 60 mg/m^2^ doxorubicin on the second day and 2 g/m^2^ ifosfamide for five days. The patient appeared to be disease-free at 14 months. No evidence of recurrence was identified on the clinical or imaging examinations during the 14-month follow-up period. However, the patient succumbed to multi-organ metastases 12 months later.

## Discussion

DSRCT is a rare and aggressive, malignant tumor. The disease most commonly presents with a multinodular growth on the serosal surfaces, including the peritoneum (3) and the pleura. DSRCT of the abdominal cavity has also been frequently documented. Extra-abdominal DSCRTs, particularly those arising in the genital system, are rare. Only one abdominal DSRCT with scrotal metastases has been previously reported and sporadic cases have occurred in the paratesticular region (4,5). The present study provides the first case of DSRCT arising in the testis to be reported in the English literature.

Morphologically, DSRCT is characterized by nests of mitotically active, small, round, blue cells that are proliferating in a cellular fibrous stroma. Immunoreactivity indicates a blastomatous cell of origin with a polyphenotypic appearance. The immunohistochemical characteristics of DSRCT exhibit the typical immunophenotype, consisting of positivity for keratin, vimentin, desmin and NSE. The differential diagnosis of DSRCT is fairly broad and includes tumors such as Ewing sarcoma, neuroblastoma, Wilms tumor, rhabdomyosarcoma, small cell carcinoma and lymphoma. A definite diagnosis may only be achieved with a demonstrated multidirectional differentiation and coexpression of epithelial, mesenchymal and neural antigens in the same cell (6). The presence of perinuclear dot-like immunostaining with desmin strongly suggests a diagnosis of DSRCT (7).

Clinically, laboratory test results are non-contributory. Several studies have described DSRCT with elevated serum CA 125 and lactic dehydrogenase levels in certain cases (8,9). These may be useful markers for DSRCT and may allow a clinician to monitor the progress of the treatment.

A DSRCT is characterized by a growth on the serosal surface. Certain studies have described an extra-abdominal location as the primary site of the tumor (10). To the best of our knowledge, the extra-abdominal testicular location of the tumor of the current case has not been previously reported. The most frequent presenting complaint for DSRCT is a painless mass and metastasis. This suggests that DSRCT should be included in the differential diagnosis of other testicular germ cell cancers.

The most common CT feature that has been reported for DSRCT is the synchronous presence of multiple abdominal masses and scrotal nodules without a clear organ of origin (5). Large homogeneous soft-tissue masses that nearly fill the entire peritoneal space have been identified. DSRCT should be suspected in young males that present with multiple bulky heterogeneous soft-tissue masses (11,12). A differential diagnosis of DSRCT arising in the testis is difficult to distinguish from other germ cell carcinomas according to the imaging features.

Despite aggressive treatment, the survival rate of patients with DSRCT remains poor. The optimum treatment remains to be determined. According to a literature review and our experience, a surgical resection, followed by aggressive chemotherapy is recommended as a treatment for DSRCT. Aggressive multimodality therapies with immunotherapy or bone marrow ablation, and dose-intensive chemotherapy with autologous peripheral blood stem cell support, may provide potential benefits for patients with DSRCT and be promising new treatment approaches (13). The survival rate may be improved using high-dose multi-drug combination chemotherapy followed by an aggressive surgical resection, radiotherapy and myeloablative chemotherapy with stem cell rescue (14).

In conclusion, primary DSRCTs of the testis are rare. In the present case, there were no specific characteristics in the clinical symptoms or the imaging studies, which resulted in a difficulty in diagnosing DSRCT. To date, no standard treatment has been established and the prognosis of affected patients is poor. Further investigation is required to identify the optimum treatment approach.
